# Ectopic Pleural Parathyroid Adenoma Causing Recurrent Primary Hyperparathyroidism

**DOI:** 10.7759/cureus.25101

**Published:** 2022-05-18

**Authors:** Jasleen Kaur, Tyler Drake

**Affiliations:** 1 Endocrinology, Diabetes and Metabolism, University of Minnesota School of Medicine, Minneapolis, USA; 2 Endocrinology, Diabetes and Metabolism, Minneapolis Veterans Affairs Health Care System, Minneapolis, USA

**Keywords:** parathyromatosis, pleural adenoma, ectopic, persistent, primary hyperparathyroidism

## Abstract

Ectopic parathyroid glands can cause primary hyperparathyroidism (PHPT) in up to 16% of cases. These can lead to recurrent/persistent PHPT, and multiple cases have been described in the literature. We present a case of a pleural parathyroid adenoma leading to persistent PHPT. The ectopic location of the parathyroid glands is a result of their migration during development and is next to tissues they share a common embryologic origin with. The pleural location of the parathyroid adenoma in our patient was highly unusual, as the pleura have an embryologically distinct origin from the parathyroids. This was likely the result of incomplete removal of a previous mediastinal adenoma or seeding of abnormal cells in the surrounding tissue due to capsular breach (parathyromatosis). These can lead to recurrent/persistent PHPT. Parathyromatosis can lead to the presence of adenomas in highly unusual locations, making diagnosis and treatment difficult. To our knowledge, this is the first reported case in the literature of pleural adenoma causing persistent PHPT.

## Introduction

Primary hyperparathyroidism (PHPT) occurs when one or more parathyroid glands start producing parathyroid hormone (PTH) autonomously. The most common presentation is the incidental discovery of mildly elevated calcium levels during routine blood work [1]. The most common cause is parathyroid adenoma (≈85% of cases), followed by parathyroid hyperplasia (≈15%), and, very rarely, parathyroid carcinoma (<1%) [1]. Parathyroid glands can be supernumerary (>four glands as a result of fragmentation during development) in about 13% of the cases; most of these glands are within the thymus [2]. Parathyroid adenomas are solitary in a vast majority of cases, but they could be located in ectopic locations in approximately 16% of the cases [3]. These supernumerary and ectopic parathyroid glands can result in recurrent or persistent hyperparathyroidism. The inferior parathyroids glands are more likely to be in an ectopic location due to their prolonged course of descent during development [4]. The ectopic inferior parathyroid glands are most commonly present within the thymus (≈30%), the anterior mediastinum (≈20%), the thyroid (≈20%), or the thyrothymic ligament (≈15%) [4-5]. Ectopic superior parathyroids are most common in the tracheoesophageal groove (≈45%), retro-esophageal area (≈20%), or posterior mediastinum (≈15%) [4-5]. Herein, we present an unusual case of persistent PHPT due to a large pleural parathyroid adenoma.

## Case presentation

**Figure 1:**
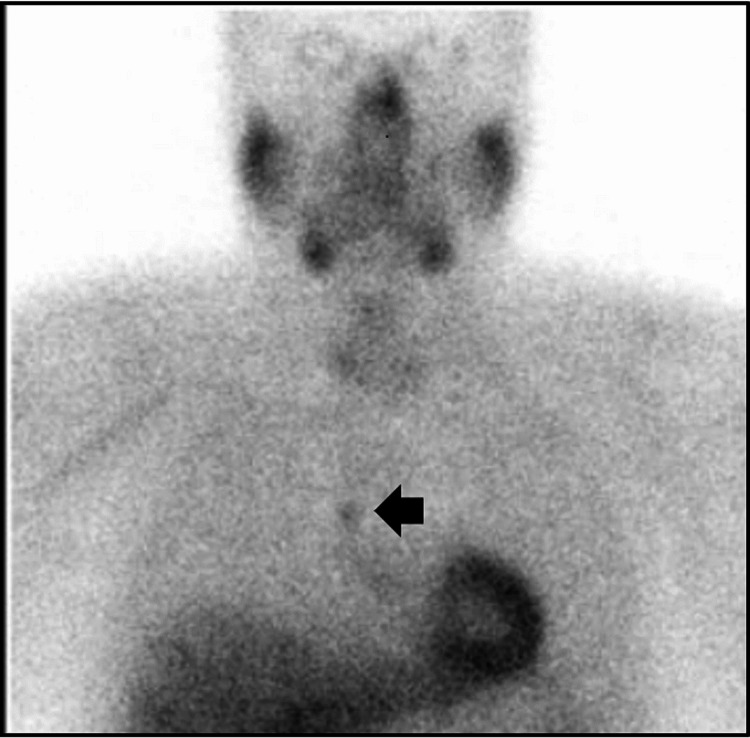
Sestamibi scan, December 2010

The patient is a 68-year-old male with a history of hypertension, hyperlipidemia, benign prostatic hyperplasia, and obesity. He presented in 2010 with incidental hypercalcemia. Further workup found elevated PTH level (Table 1) consistent with primary hyperparathyroidism. He met surgical criteria with serum calcium >11 mg/dL. Sestamibi scan localized the parathyroid adenoma to the anterior mediastinum (Figure 1; black arrow).

**Table 1:** Laboratory evaluation for primary hyperparathyroidism

DATE	Calcium (8.5-10.1 mg/dL)	iPTH (11.1-79.5 pg/mL)	Albumin (3.4-5.0 g/dL)	Phosphorus (2.5-4.9 mg/dL)	eGFR (>60 mL/min/1.7m^2^)
2/18/2011	11.8	141.0	4.4	-	
First surgery (7/8/2011)					
7/9/2011	9.5		3.8		66
5/14/2015	11.3	110.3	3.8	2.5	>60
Second surgery (5/15/2015)					
5/15/2015	9.7	18.1	-	2.1	56
1/25/2017	10.2	90.5	3.8	3.3	56
9/28/2018	11.6	118.7	3.9	1.9	>60
8/26/2020	12.1	164.5	4.2	2.4	>60
Third surgery (8/27/2020)					
8/27/2020	-	45.6	-	-	-
8/28/2020	10.3	-	-	-	>60
9/24/2020	10.5	-	-	-	>60
7/23/2021	11.1	74.6	3.8	3.0	>60
11/17/2021	11.6	-	3.8	-	>60
2/17/2022	10.7	-	3.7	2.9	>60
3/2/2022	-	43.0	-	-	-

**Figure 2:**
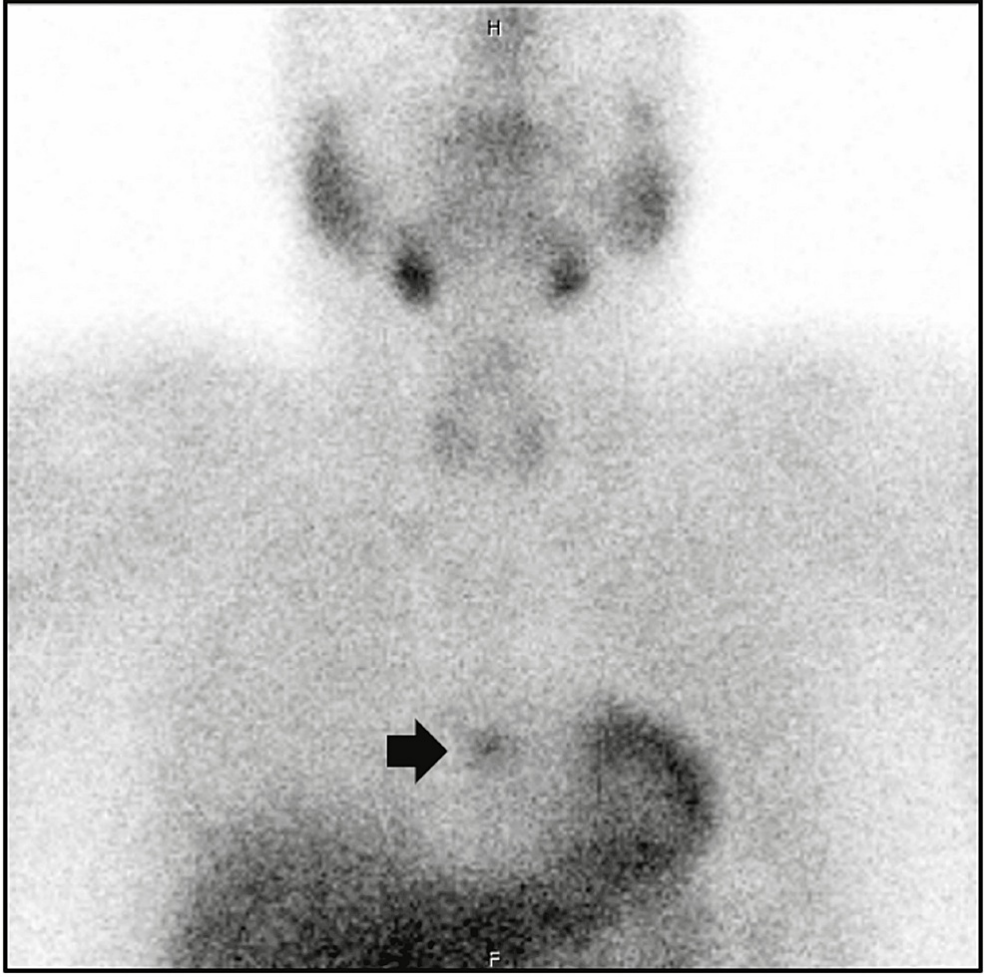
Sestamibi scan, April 2015

Right-sided video-assisted thoracoscopic surgery (VATS) was performed for resection of the mediastinal parathyroid adenoma. Pathology revealed hypercellular parathyroid tissue with adjacent involuted thymic tissue. Serum calcium levels normalized after surgery (Table 1). During follow-up, the serum calcium levels were again elevated in 2013 with high PTH levels. The Sestamibi scan localized an adenoma to the anterior mediastinum again (Figure 2; black arrow). CT chest with contrast demonstrated an oval mass of 10 x 3.5 x 13 mm in the area corresponding to the Sestamibi findings, adjacent to the heart. There was a concern for possible incomplete resection of prior parathyroid adenoma leading to recurrent hyperparathyroidism.

**Figure 3:**
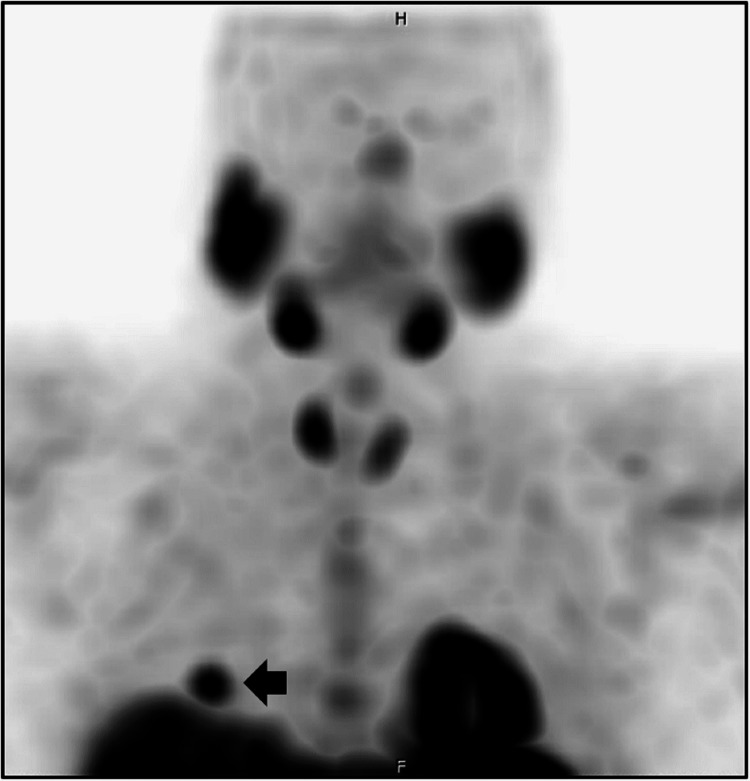
Sestamibi scan, September 2019

A decision was made to proceed with resection via sternotomy. The aim was to remove all mediastinal fat to ensure the removal of the adenoma. Intraoperatively, the adenoma was found to be adherent to the upper lobe of the right lung. So, wedge resection of the right upper lobe was performed. The parathyroid adenoma was confirmed on frozen section intraoperatively. Final pathology reported the mass to be parathyroid adenoma measuring 1.2 x 0.9 x 0.4 cm and weighing 0.404 grams. Serum calcium and PTH levels normalized after surgery. During subsequent follow-up, serum calcium levels started rising again in 2017 with elevated PTH levels. Sestamibi scans were repeated in 2017 and 2018 and did not localize a parathyroid adenoma. In 2019, the Sestamibi scan revealed an area of increased uptake noted in the posterior aspect of the lower right lung (Figure 3; black arrow). Since Sestamibi is a nonspecific tumor marker, this uptake raised suspicion of malignancy. CT scan of the chest then revealed a nodule measuring 14 x 5 mm corresponding to the area of Sestamibi uptake. A CT-guided biopsy of the pleural nodule was performed and showed fragments of oxyphilic parathyroid type tissue, confirming recurrent primary hyperparathyroidism.

He then underwent right-sided VATS for resection of pleural parathyroid adenoma. Pathological findings showed hypercellular parathyroid tissue consistent with an adenoma weighing 3.945 grams. Postoperative PTH normalized, but serum calcium remained mildly elevated (Table 1).

Follow-up

Serum calcium levels again began rising in 2021 with high normal PTH (Table 1). A subsequent Sestamibi scan in 2021 failed to localize a parathyroid adenoma. He is doing well clinically and asymptomatic from his hypercalcemia. He is now being monitored clinically and biochemically every six months for his persistent/recurrent primary hyperparathyroidism, knowing he will likely require a fourth surgery once imaging localizes another adenoma.

## Discussion

Persistent PHPT is defined as failure to attain normocalcemia within six months of surgery [6]. Recurrent PHPT is defined as the redevelopment of hypercalcemia after at least six months of normal calcium levels after surgery [6]. These cases commonly occur as a result of ectopic parathyroid adenomas. These adenomas tend to lie next to tissues with whom they share their embryologic origin [4]. The inferior and superior parathyroid glands arise from the third and fourth pharyngeal pouches respectively, these pouches develop due to lateral migration of the endoderm and present as outpouchings of the primitive pharynx [4]. In our patient, the first and second adenomas were found in the anterior mediastinum, where the structures have embryologic origins similar to the inferior parathyroids. But the third time, the adenoma was found next to the right posterior parietal pleura, which has a different embryologic origin. The parietal and visceral pleura of the lungs arise from the somatopleuric and splanchnopleuric layers of the mesoderm [7]. To the best of our knowledge, this is the first case of PHPT secondary to pleural parathyroid adenoma.

Despite significant improvements in imaging modalities and surgical techniques, persistent or recurrent PHPT continues to occur in 2.5-5% of cases of PHPT [6]. The causes of recurrent or persistent PHPT include failure to locate an incident adenoma, dormant second parathyroid adenoma (also known as subordinate adenoma), missed parathyroid hyperplasia, incomplete removal of a single parathyroid adenoma, parathyromatosis (breakage of capsule leading to seeding of abnormal parathyroid tissue in surrounding soft tissue/structures), or parathyroid carcinoma [8-10].

PHPT due to ectopic adenomas tends to be associated with higher serum calcium levels, larger adenomas, and a high incidence of hyperparathyroidism-associated bone disease [11]. The mean weight of parathyroid adenomas is 553.7 ± 520.5 mg [12]. In our patient, the pleural adenoma was 17 mm in the largest dimension, weighed 3.945 grams or 3945 mg, and was associated with the highest serum calcium level of 12.1 mg/dL. Interestingly, parathyroid carcinomas are also associated with larger and metabolically more active masses [13]. More than 80-85% of these tend to be >3 cm in size and are associated with serum calcium >12.0 mg/dL [13]. Pulmonary metastasis can be present in up to 40% of the cases [14]. These lung metastases have been shown to produce sufficient PTH to lead to severe hypercalcemia [14]. In our patient, there was a concern for parathyroid carcinoma, given the localization to the right pleural space with serum calcium >12.0 mg/dL and a larger lesion. But this was ruled out with a benign pathology showing hypercellular parathyroid cells with similar morphology to his previous resections.

In our patient, the second adenoma was localized to the same location as the first one on a parathyroid Sestamibi scan and CT of the chest in the anterior mediastinum. This did raise concern for incomplete resection. Whether this was due to partial resection or parathyromatosis leading to the seeding of abnormal parathyroid cells in the surrounding soft tissue in the mediastinum remains unclear. The second adenoma was adherent to the lung, necessitating resection of the upper lobe of the right lung and excision of the remaining mediastinal fat. For the third adenoma, the location of the pleural-based adenoma is highly unusual, and we hypothesize seeding of the parathyroid cells intraoperatively from either the first or second surgical resections, causing parathyromatosis. While parathyroid carcinoma was considered given this location, this was ruled out by the mild elevations in PTH, prolonged time course, and benign-appearing pathology. Unfortunately, the patient did not have normalization of calcium levels after his most recent surgery suggesting one or more areas of additional seeding, which have remained undetectable on further imaging.

## Conclusions

Ectopic parathyroid glands are important cases of recurrent/persistent HPTH. An understanding of the embryologic origins of these glands is necessary. During surgery, a complete resection of the adenoma with careful attention to preserve the integrity of the capsule is needed to prevent incomplete removal or seeding of abnormal cells in the surrounding tissue. These can lead to recurrent/persistent HPTH, which can be difficult to cure.
